# Digital solutions for primary stroke and cardiovascular disease prevention: A mass individual and public health approach

**DOI:** 10.1016/j.lanwpc.2022.100511

**Published:** 2022-06-30

**Authors:** Valery L. Feigin, Rita Krishnamurthi, Alexander Merkin, Balakrishnan Nair, Michael Kravchenko, Shabnam Jalili-Moghaddam

**Affiliations:** a National Institute for Stroke and Applied Neurosciences, School of Clinical Sciences, Auckland University of Technology, New Zealand; b Institute for Health Metrics Evaluation, University of Washington, Seattle, USA; c Research Centre of Neurology, Moscow, Russia

There is evidence that if the current trend in the non-communicable diseases (NCDs) burden continues, the UN Sustainable Development Goal (SDG) target 3.4 - to reduce premature mortality from NCDs by a third by 2030 - will not be met in most countries.^1^ As stroke and other cardiovascular diseases (CVDs) are the leading causes of death and disability worldwide contributing the most to the NCDs mortality in nearly every country, SDG target 3.4 cannot be achieved without substantial reduction in the burden of stroke and other CVDs. Although highly preventable (up to 90%),^2^, ^3^, ^4^ their burden has greatly increased over the last three decades, disproportionally affecting low- to middle- income countries.^2^^,^^5^ The continuously increasing number of persons affected by stroke and other CVDs with a tendency towards affecting younger persons, along with increased prevalence of CVD risk factors (e.g., obesity, diabetes mellitus, peripheral artery disease, hyperglycaemia, atrial fibrillation)^1^^,^^2^^,^^5^ strongly suggests that existing primary stroke and CVD prevention strategies are not sufficiently effective.^6^ Therefore, a novel, more effective approach to reduce the incidence of stroke and other CVDs is urgently needed.

The individual-focused high-risk CVD prevention approach has been the most prevalent over the past 30 years.^6^ Evidence supports the implementation of more inclusive interventions for persons at any level of increased risk of stroke and CVD, that reduce health inequities and address risk factors in countries and communities who need it most.^7^ This holistic approach has been endorsed by the World Stroke Organization^8^ and World Heart Federation in their recent joint Declaration^9^ and other publications on stroke and CVD prevention.^7^^,^^10^, ^11^, ^12^, ^13^, ^14^

The main problems in effective stroke and CVD prevention strategies on the individual level relate to: (a) lack of public knowledge about stroke/CVD warning signs and risk factors or an individual's personal risk of having a stroke/CVD and hence a lack of motivation on the side of individuals/lay persons to control their modifiable risk factors; (b) absence of evidence-based primary stroke/CVD digital tools to empower lay persons, and arm health professionals with a patient-centred approach to support primary prevention decision-making and improve primary stroke/CVD guidelines uptake; and (c) inadequate time during clinical encounters for clinicians to provide effective person-centered primary prevention recommendations and guidance. In addition, preventative strategies targeting high CVD risk individuals are inherently limited to the high-risk individuals, while the majority of stroke and acute CVD disease occur among persons with low- to moderate CVD risk.^6^^,^^15^^,^^16^

To address these issues, the National Institute for Stroke and Applied Neurosciences of Auckland University of Technology, New Zealand, in collaboration with national and international experts in stroke and CVD, including general practice clinicians, neurologists, public health experts, and consumer groups, has developed and validated two unique award winning digital tools: (1) the free Stroke Riskometer™ app for individual use which has received a World Stroke Organization Award, McDiarmid Medal of the Royal Society of New Zealand, Australasian Stroke Society Award, and (2) the PreventS-MD™ webapp for health professionals based on the Stroke Riskometer algorithm which won the WHO Western Pacific Innovation Challenge Award.^17^, ^18^, ^19^

The stroke prediction algorithm in both tools was derived from the Framingham Stroke Risk Score prediction algorithm^20^ and enhanced to include several additional major (mainly lifestyle) risk factors shown to be important for stroke and CVD occurrence.^3^^,^^4^ Inclusion of additional risk factors is also justified from a public health perspective because their control allows reduction of the risk of not only stroke and CVD but also other major NCDs sharing the risk factors, such as diabetes mellitus, renal vascular disease, vascular dementia and some types of cancer. The risk factors included are: age (range 20–93 years), sex, self-identified race/ethnicity (African, Arabian/Persian, Chinese, Indian, Japanese, Latin American/Hispanic, Malay/Indonesian, Māori, Other Asian, Pacific Islander, White/European, Other), systolic blood pressure, smoking, diet, physical activity, presence of heart disease, family history of stroke and/or heart disease, use of blood pressure lowering medication, diabetes mellitus, height and weight (Body Mass Index is calculated automatically), psychosocial stress, history of traumatic brain injury, mild cognitive problems or dementia.

PreventS-MD™ is a risk assessment, patient management and decision support software for health care professionals (HCPs) that can be connected with or embedded into the existing electronic patient record management systems of healthcare providers or used independently. It is designed specifically for HCPs to optimise patient-specific risk factor management, to monitor the control of stroke/CVD risks and to facilitate discussions that can motivate patients to adopt healthy lifestyle interventions (Figure 1). The PreventS-MD™ webapp does not provide specific medical evaluation, diagnosis or treatment, but rather equips HCPs with a reliable and validated tool for risk assessment that ensures better identification of patients at risk of developing stroke and/or acute coronary syndrome. It also provides clinicians with pre-completed evidence-based but editable patient-tailored recommendations for primary stroke and CVD prevention, which are based on internationally recognised primary stroke and CVD prevention guidelines. By communicating with electronic patient record management systems, the PreventS-MD™ webapp allows for semi-automated collection of information on stroke and CVD risk factors during subsequent clinical encounters. In most healthcare systems, stroke-specific risk assessment and desicion support management tools are not available to HCPs as such, and this tool addresses an important healthcare information gap. Stroke-specific risk assessment/management systems are currently not available to HCPs in most countries.

**Figure 1:**
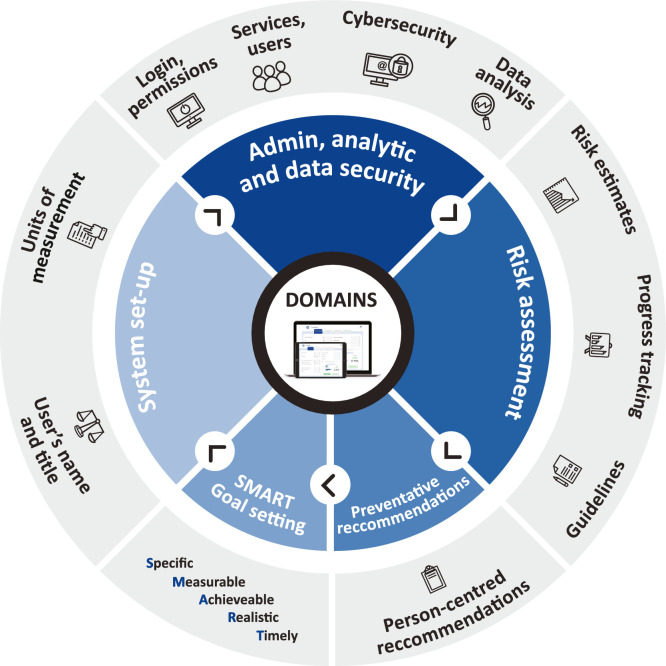
Main domains and functionality of PreventS-MD™.

In the PreventS-MD™ webapp we included an acute coronary syndrome prediction algorithm that was also derived from the Framingham Heart Study.^21^ In addition to the risk factors used in the Stroke Riskometer app for estimating the risk of stroke, we included two additional risk factors (total cholesterol and high-density lipoprotein cholesterol) required for estimation of the risk of acute coronary syndrome. PreventS-MD™ also includes an option for the treating physician to set a ‘recommended systolic blood pressure for the individual’ as one of the goal settings for the individual. In addition, unlike any other digital applications for stroke and CVD predictions,^17^ these cognitive behavioural theory^22^ based tools provide estimates of not only absolute 5 and 10-year risks but also relative risk of stroke and acute coronary syndrome for persons of different race/ethnicity aged 20–93 years. Risk of acute coronary syndrome is estimated only for persons aged 30–74 years, without history of cardiovascular disease and when data on total and high-density lipid cholesterol are available. This way of communicating risk assessment and management can lead to increased motivation for behavioural change.^23^^,^^24^ Furthermore, both digital tools contain graphical easy-to-understand visualisation of the risks and their trends over time, ‘ideal’ levels of risk factors for the individual, goal setting options, medication reminders, and evidence-based recommendations for primary and secondary stroke and CVD prevention.

For individuals willing to update their risk data on the Stroke Riskometer app using the PreventS-MD™ screening results, there is an option to transfer the PreventS-MD™ data to the Stroke Riskometer app, using a QR code. It is also possible for nurses in outpatient clinics or hospital wards to pre-enter variables in PreventS-MD™ and wirelessly transfer the data to the doctor's office computer, thus saving the treating physician's time on data entry when they assess the individual. These two digital tools are complementary to each other and ideally should be used in tandem (Figure 2) – HCPs use the PreventS-MD™ webapp on their office computers and/or portable devices while lay persons and community workers use the Stroke Riskometer mobile app on their smartphones and portable devices. Based on the results of the pilot randomised controlled trials in New Zealand^23^^,^^25^ and Malaysia,^26^ it is expected that the wide use of the Stroke Riskometer™ app and PreventS-MD™ over just 5 years could reduce stroke incidence in the world by up to 20%. Two large full-scale randomised controlled trials are currently underway in Australia, New Zealand (ACTRN12621000211864) and Brazil^27^ to confirm the efficacy of these two digital tools as compared to usual care.

**Figure 2:**
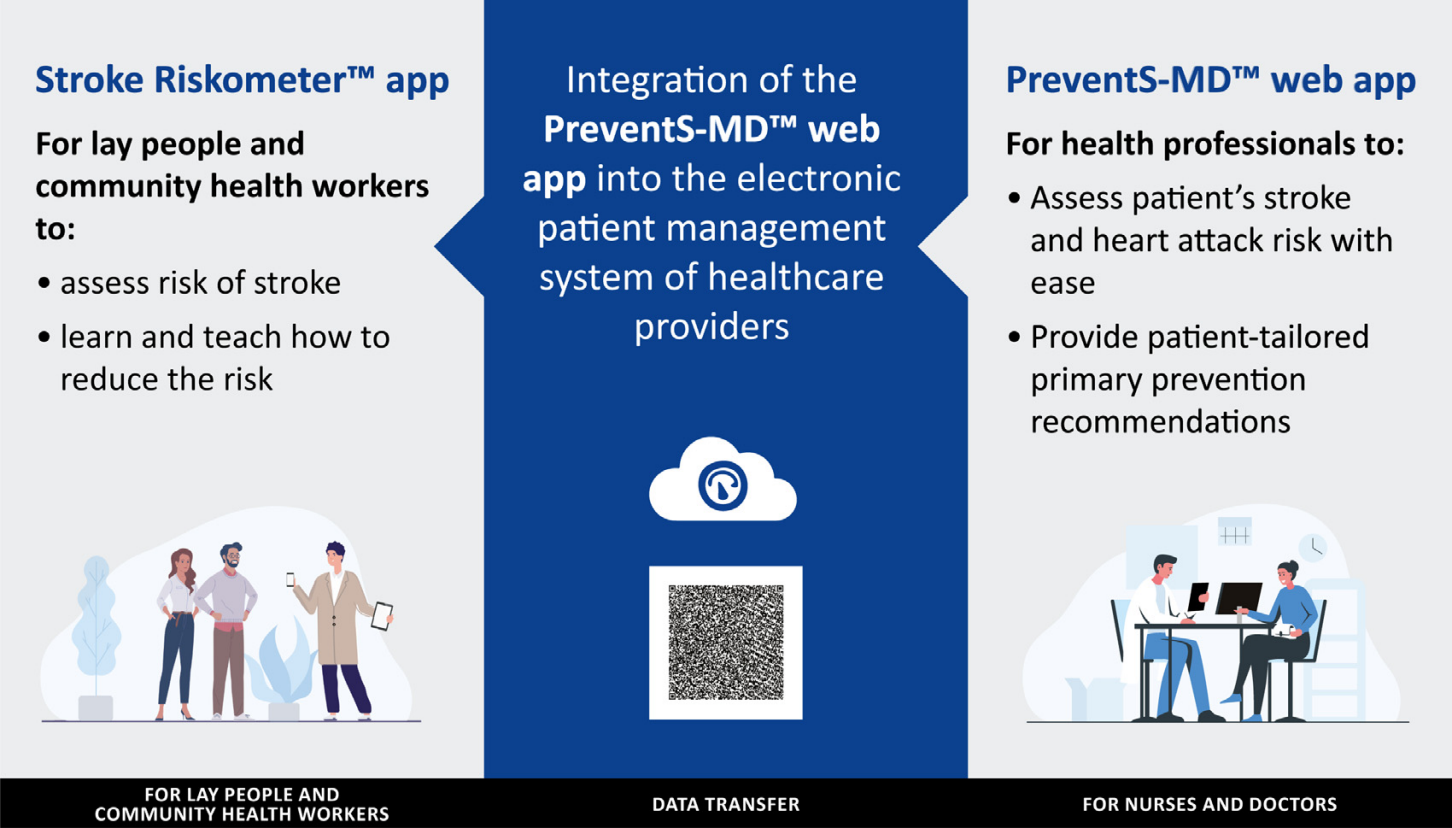
Integrative and complimentary use of the Stroke Riskometer™ app and PreventS-MD™ for primary prevention of stroke, cardiovascular and other major non-communicable diseases on the individual level.

The other important feature of PreventS-MD™ is that it has a built-in analytical module allowing administrators of the healthcare organisation to analyse medical and socio-demographic characteristics of persons screened by PreventS-MD™ and efficacy of the stroke and CVD prevention interventions at different levels of services (individual clinician, local, regional or national healthcare service) over a given time period. While ensuring protection of data privacy (including data access, management, storage and interpretation) and sovereignty (collection, ownership) of the healthcare provider, this analytical tool and data may also be of value for researchers, provided that local ethics committee and other requirements are met.

Results of pilot randomised controlled trials^23^^,^^26^ showed good acceptability and preliminary efficacy of the Stroke Riskometer™ app in reducing the risk of stroke/CVD and increasing awareness about stroke. Similarly, preliminary results from the recent PRIME International Study revealed good System Usability Scale^28^ score (*M* = 80.22; 95% confidence interval [76.96, 83.47]), high level of feasibility and satisfaction (usefulness ranging from 88 to 98%) with the PreventS-MD™ webapp by HCPs from 27 countries and individuals at risk of stroke/CVD. Being already available for free in 19 languages (Bengali, Brazilian-Portuguese, Bulgarian, Chinese (Mandarin), Croatian, Czech, English, French, German, Greek, Hindi, Italian, Malay, Nepali, Portuguese, Russian, Spanish, Swedish, Thai), the Stroke Riskometer™ app is available to 5.3 billion persons in their native languages. It has so far been downloaded by more than 200,000 persons from 78 countries. Translated versions of the app have been adapted by local translators, who themselves are experts in stroke, to different cultural contexts in particular regions, thus allowing its adaptivity to different social, economic and cultural contexts regionally and globally.^19^ There are also significant prospects for scalability of the PreventS-MD™ as the only currently available - and adaptable to different settings - digital decision support making tool for HCPs to enhance their primary stroke and acute coronary syndrome activity.^17^ It is suggested that the wide use of the Stroke Riskometer^TM^ app by lay persons (about 84% of persons in the world have smartphones^29^) and the PreventS-MD™ webapp by HCPs (the use of electronic patient management systems by office-based physicians is expanding rapidly^30^ already reaching 86% in the USA^31^), would foster social inclusion, reinforce the achievements of the SDGs (especially SDG 3; to ensure healthy lives and promote well-being for all at all ages) and facilitate bridging the gap in universal health coverage for the poorest billion persons in the world.^32^ There are several large studies currently underway to establish safety, usability, efficacy (compared to usual care), data privacy and security, as well as the acceptability, and barriers to implementation of these digital tools.

The Stroke Riskometer™ app has been endorsed globally by several non-government organisations, including the World Stroke Organization, World Heart Federation, World Federation of Neurology, European Stroke Organisation and a number of national stroke organisations (e.g., the Australian Stroke Foundation, French Neuro-Vascular Society, and Chinese Stroke Society). In the recent Policy View paper of the World Stroke Organization expert group published in *The Lancet Public Health* both the Stroke Riskometer™ app and the PreventS-MD™ webapp have been recommended for global use.^7^ As stroke risk factors included in the Stroke Riskometer™ app and PreventS-MD™ are also common for other major NCDs, such as ischaemic heart disease, renal disease, dementia, type II diabetes mellitus, and even some type of cancer, controlling these risk factors could also facilitate reducing the burden from all these NCDs,^7^^,^^8^ thus saving the lives of millions of people around the globe.

## Contributors

VLF conceptualised the idea, wrote the original draft, and edited the manuscript to incorporate co-authors’ inputs. All other co-authors provided input on evidence to include, reviewed and edited draft manuscripts. All authors revised the paper critically for intellectual content and approved the final version.

## Role of the funding source

No external funding was used for this research.

## Declaration of interests

The authors state that free Stroke Riskometer™ app and PreventS-MD™ webapp are owned and copyrighted by AUT Ventures Ltd, Auckland University of Technology, New Zealand.
